# Cognitive Reserve as a Useful Concept for Early Intervention Research in Multiple Sclerosis

**DOI:** 10.3389/fneur.2015.00176

**Published:** 2015-08-20

**Authors:** James F. Sumowski

**Affiliations:** ^1^Kessler Foundation, West Orange, NJ, USA; ^2^Rutgers New Jersey Medical School, Newark, NJ, USA

**Keywords:** multiple sclerosis, cognitive reserve, rehabilitation, cognition, memory

## Cognitive Impairment in Multiple Sclerosis

Multiple sclerosis (MS) is a lifelong progressive neurologic disease typically diagnosed between ages 20 and 40 years: a time when persons are striving to accomplish normative goals of young adulthood (e.g., establishing a career). More than half of MS patients suffer cognitive decline [for review, see Ref. (1)] especially memory problems and cognitive inefficiency (e.g., slowed processing speed, difficulty multi-tasking).

## Clinico-Pathologic Dissociation

There is great variability in cognitive status across MS patients, even among patients with similar patterns of disease burden/progression (2, 3). This is evidenced in part by the relatively modest/incomplete correlation between MS disease burden (e.g., T2 lesion volume, cerebral atrophy) and cognitive functions, whether studied cross-sectionally [e.g., Ref. (2)] or longitudinally [e.g., Ref. (3)]. That is, some MS patients are better able to cope with disease burden without cognitive deficits. [Note: there is an important and advancing literature on the relationship between cognition and MRI parameters in persons with MS [e.g., Ref. (4–6)], although a thorough review of this literature is beyond the scope of this opinion piece. In each case, however, the relationship between disease burden and cognitive outcomes remains incomplete.] This dissociation between disease burden and cognitive outcome is common in other neurologic diseases as well, including Alzheimer’s disease (AD) (7–9). Indeed, some persons accumulate substantial AD neuropathology (e.g., beta-amyloid) without dementia, whereas other persons suffer dementia at comparable or even lower levels of pathology (8, 9). These observations have motivated the question: how are some people better able to withstand neurologic disease burden without cognitive impairment?

## Importance of Prediction and Early Intervention to Prevent Cognitive Decline

Systematic reviews report little-to-no efficacy of pharmacological (10) and behavioral (11) treatments for memory impairment in MS patients. As such, the best treatment of cognitive impairment in MS may be the proactive prevention of cognitive decline in the first place. Similarly, treatments for memory impairment in persons with AD have proven largely ineffective, and research has recently shifted toward very early pre-clinical intervention to prevent the onset of dementia (which may represent a point of no return). The science and clinical practice of early intervention/preventative medicine hinges on our ability to accurately identify patients at greatest risk for future cognitive decline or dementia. Targeted enrollment of at-risk patients into early intervention trials will improve statistical power, because beneficial effects of early treatment can only be observed if the non-treatment group declines. Enrolling at-risk patients ensures that there will be adequate cognitive decline for the early intervention to moderate. Clinically, at-risk patients could be targeted for early interventions to help prevent future cognitive decline, and earlier treatment takes advantage of the brain’s capacity for plastic reorganization, which is ostensibly greater at younger ages. Finally, if risk and protective factors are modifiable, then knowledge of such factors can inform treatment decisions and/or counseling of patients regarding healthy life choices. First, however, we need to advance our ability to accurately identify MS patients at greatest risk for future cognitive decline.

## Cognitive Reserve Against Cognitive Decline

The disconnect between disease burden and cognitive status (i.e., differential cognitive decline) is explained in part by the cognitive reserve hypothesis (12–14), which posits that enriching life experiences protect against cognitive decline in the face of aging and neurologic disease, likely due to greater capacity and efficiency of neural networks (15, 16). Support for the cognitive reserve hypothesis has come from evidence that older adults with a history of greater educational or occupational attainment (17, 18) or engagement in cognitively stimulating leisure activities (19–21) are at reduced risk for dementia. Importantly, the later work showed that cognitive leisure activity (e.g., reading, hobbies) among healthy elders reduced risk for incident dementia in the future, suggesting that consideration of such behaviors in elders may be a useful predictor of future cognitive decline. Note also that engagement in intellectually enriching activities moderates/attenuates the deleterious effect of AD neuropathology on cognitive status in elders (22, 23). Taken together, there is now amble observational evidence within the aging literature that lifetime intellectual enrichment and current cognitive leisure activity lower risk for dementia.

Work by myself and others has extended the cognitive reserve hypothesis to MS [for review, see Ref. (14)], showing that MS patients with greater education (24–27) and literacy/vocabulary (estimated with vocabulary) (28–31) are protected against disease-related cognitive inefficiency and memory problems. We have also shown, however, that cognitive leisure activity (e.g., reading, hobbies) contributes to cognitive status in MS patients independently of lifetime enrichment (estimated with vocabulary) (32), and that engagement in such leisure activities during early adulthood moderates/attenuates the negative effect of disease burden (T2 lesion volume) on current cognitive status in MS patients (33). Others have also shown a benefit of leisure activity against cognitive impairment in MS (34–36). Longitudinal research on reserve against cognitive decline has been more limited; however, Benedict and colleagues have shown that greater intellectual enrichment protects against decline in cognitive efficiency over nearly 5 years (24), and we have shown that enrichment is protective against decline in cognitive efficiency and memory over 4.5 years (31). Longitudinal research highlights the potential clinical importance of considering a patient’s level of lifetime enrichment (easily assessed with vocabulary knowledge), which may be a useful predictor of future cognitive decline (thereby helping to identify at-risk patients).

**Figure 1:**
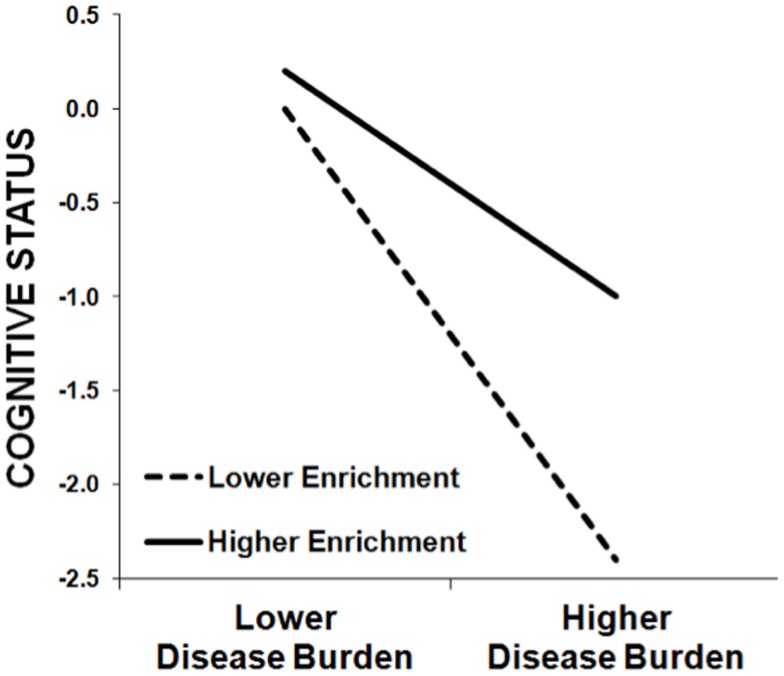
**This schematic demonstrates the protective effect of enrichment against cognitive impairment in MS patients, whereby the negative relationship between cognitive status (*y*-axis) and MS disease burden (*x*-axis) is stronger among patients with lower enrichment (dashed line) relative to patients with higher enrichment (solid line)**. That is, higher enrichment attenuates the negative effect of MS disease burden on cognitive status. (Note that this schematic was not derived from actual data, but instead represents the typical pattern of results we have observed previously.)

On the one hand, we are not surprised that education predicts cognitive outcomes, as such correlations are observed in healthy persons as well. Importantly, however, the theory of cognitive reserve is not based on this main effect of enrichment; rather, cognitive reserve is instantiated in a moderation/interaction. Higher enrichment moderates/attenuates the negative relationship between a disease-related variable (e.g., lesion volume, cerebral atrophy) and a cognitive outcome (e.g., memory). As such, the negative impact of disease burden on cognition is actually greater in persons with lower enrichment than persons with higher enrichment (see Figure 1). In fact, we have previously demonstrated that the amount of variance in cognitive outcomes accounted for by disease burden (e.g., cerebral atrophy) actually varies based on the educational attainment of the MS sample, with a stronger relationship between disease burden and cognitive outcomes in samples with lower education (28). The theory of cognitive reserve posits that greater intellectual enrichment protects persons with MS from the negative impact of disease burden on cognition, leading to different trajectories of cognitive decline over time [e.g., Ref. (31)].

## Brain Reserve Against Cognitive Decline

Separate from the cognitive reserve hypothesis, the theory of brain reserve capacity (37) proposes that cognitive impairment emerges when brain volume falls below a critical albeit unspecified threshold. This theory has been supported by observations that elders with larger head circumference or intracranial volume [proxies of the brain’s maximal lifetime brain growth (MLBG)] are at reduced risk for cognitive decline or dementia (38, 39). MLBG is considered a proxy of neuronal/synaptic count [see Ref. (40)], and greater neuronal/synaptic count may (a) be linked to more robust neural networks resistant to disease-related disruption and/or (b) provide more potential degrees of freedom for the brain to plastically reorganize in the face of aging or disease-related challenges. We have recently shown that larger MLBG lowers risk for cognitive impairment in MS. Specifically, larger MLBG (estimated with intracranial volume) moderated/attenuated (a) the deleterious link between MS disease burden (e.g., T2 lesion volume) and cognitive efficiency in a cross-sectional sample (33), and (b) decline in cognitive efficiency over 4.5 years in a longitudinal sample (31). Note that MLBG was unrelated to memory function within our MS samples, and closer inspection of the aging/AD literature suggests that MLBG is protective against cognitive inefficiency rather than episodic memory deficits [for discussion, see Ref.(33)]. Note that our cross-sectional (33) and longitudinal (31) research showed that intellectual enrichment protects against cognitive inefficiency independently of MLBG, which is important given the robust moderate correlation between brain size and intelligence (41).

Clinical consideration of MLBG may help identify patients at greatest risk for future cognitive impairment, and such patients can be targeted for early intervention rehabilitation. Note that MLBG is almost completely heritable (42) and therefore outside of one’s current control; however, patients could be counseled regarding brain healthy choices (e.g., exercise, diet), which may prevent/slow the loss of reserve brain volume. For instance, cigarette smoking is particularly damaging for MS patients, and should be strongly discouraged (43). Also, psychological stress can exacerbate MS (44), and stress management training has reduced inflammatory MS lesions (45). Finally, adherence to pharmaceutical treatments is linked to preservation of function (46), as disease-modifying therapies are effective in reducing cerebral atrophy (preserving brain reserve) in MS patients (47). This notion of maintaining brain reserve by avoiding risk factors for neuropathology is reviewed elsewhere as the concept of “brain maintenance” in aging (48).

## Building Reserve Against Cognitive Impairment

Cognitive reserve is an appealing concept. It suggests that persons can reduce their risk of age- or disease-related cognitive decline by actively pursuing intellectually enriching lifestyles. Note, however, that evidence for the cognitive reserve hypothesis in aging and neurologic populations is almost entirely observational, thereby preventing causal statements about the protective effects of cognitive stimulation. As such, a great deal of more rigorous work is needed before we can “prescribe” specific programs of enrichment, including true experiments/randomized controlled trials of intellectual enrichment. That said, engagement in mentally stimulating activities represents a cost-effective, non-invasive way for healthy persons and MS patients to actively participate in their own cognitive health. This is non-trivial, as the unpredictable nature of MS disease often results in an external locus of control (49), leading to hopelessness and depression. MS patients should be encouraged to remain cognitively active from the time of diagnosis onward.

One important avenue for future research will be to identify modifiable neuroanatomical bases for the protective effect of reserve. We have recently linked engagement in cognitive leisure activity to larger hippocampal volume in persons with MS (35), which is consistent with the well-established effects of enrichment on the hippocampus in basic research [for review, see Ref. (50)], as well as links between enrichment and hippocampal volume in older humans (51, 52). Once we identify the neuroanatomical basis for reserve, we can use these as structural targets in early intervention work to evaluate whether preventative treatments have increased reserve. The alternative is to wait for years to see if an early intervention led to differential cognitive decline in the future, but neuroanatomical targets provide more immediate feedback on the efficacy of early interventions. Discovery of modifiable neuroanatomical bases of reserve also allows us to expand our efforts beyond cognitively based interventions (e.g., intellectual enrichment) to include other interventions/protective factors linked to the health of neuroanatomical targets. For instance, regarding the hippocampus, one of the most promising treatments across neurologic populations may be aerobic exercise training. Indeed, basic research reports strong support for the role of exercise in stimulating hippocampal neurogenesis and memory [e.g., Ref. (53)], which is being translated into humans [e.g., Ref. (54), for review, see Ref. (55)]. We have previously reported a case study linking aerobic exercise training to increased hippocampal volume, improved memory, and enhanced default network functional connectivity in MS (56), and aerobic exercise training in progressive MS patients appears promising (57). Outside of aerobic exercise training, there are many benefits of physical exercise for cognition generally in MS patients [for review, see Ref. (58)].

## Conclusion

The theory of reserve provides a useful framework for the science and clinical practice of early intervention against cognitive decline in MS patients (i.e., preventative medicine). First, consideration of a patient’s MLBG and level of lifetime intellectual enrichment may help identify patients at greatest risk for future cognitive decline. These at-risk patients can be targeted for early intervention cognitive rehabilitation, or research on such treatments. Toward this end, future research should develop and test algorithms to predict risk of cognitive decline in MS patients, which should take proxies of reserve (as well as other risk factors, e.g., smoking) into consideration. Second, intellectual enrichment programs may provide an early intervention treatment in itself; however, all existing evidence is observational, so rigorous experimental work is necessary to establish causal relationships between enrichment and protection against cognitive decline. Finally, the use of MRI or fMRI to identify neuroanatomical or functional markers of reserve will be helpful in providing measurable proxies for increased reserve as outcomes of early intervention trials. Such targets will provide an immediate evaluation of an interventions efficacy to increase reserve, which can then be validated by differential cognitive decline in the future. There is indeed much more work to be done to translate the concept of reserve into a clinically useful tool for prediction of decline, evaluation of treatment efficacy, and treatment itself for MS patients.

## Conflict of Interest Statement

This opinion was conducted in the absence of any commercial or financial relationships that could be construed as a potential conflict of interest.
